# Diagnostic Challenges in Primary Hepatocellular Carcinoma: Case Reports and Review of the Literature

**DOI:** 10.1155/2015/878763

**Published:** 2015-04-01

**Authors:** Monika Pazgan-Simon, Sylwia Serafinska, Justyna Janocha-Litwin, Krzysztof Simon, Jolanta Zuwala-Jagiello

**Affiliations:** ^1^Infectious Disease Department, Wroclaw Medical University, Wroclaw, Poland; ^2^Infectious Disease Department, Division of Infectious Disease and Hepatology, Wroclaw Medical University, Wroclaw, Poland; ^3^Department of Pharmaceutical Biochemistry, Wroclaw Medical University, Wroclaw, Poland

## Abstract

Hepatocellular carcinoma is the fifth most common malignancy and the third leading mortality cause worldwide. It typically develops secondarily to liver cirrhosis, due to hepatitis B or C infection, alcohol abuse, metabolic disease, and so forth. According to the American Association for the Study of Liver Diseases (AASLD) guidelines, which constitute diagnostic standards, the diagnosis of primary hepatocellular carcinoma (HCC) should be based on contrast-enhanced imaging. Lesion hyperenhancement should be observed throughout the arterial phase, followed by the washout during the venous phase. The diagnosis can also be based on the histopathological evaluation of liver biopsy specimen. Although the standards are clear, we often see patients with advanced HCC in clinical practice, who cannot be offered any effective treatment. Patients with chronic liver disease, presenting with inconclusive and changeable test results, constitute a separate problem. In such cases the diagnostic process is typically long-term and delayed. In this paper we present three case reports where the diagnosis could not be made promptly and the patients died as a result of a delayed diagnostic process.

## 1. Introduction

Hepatocellular carcinoma is the fifth most common malignancy (5% of all cancer cases) and the third leading cancer-associated mortality cause worldwide. It is also the most common primary hepatic malignancy (80% of liver cancer cases in adults and 35% in children) [1]. In almost all cases it is secondary to cirrhosis or other chronic liver damage. However, it may also develop without significant liver damage in children with HBV infection. HCC is the leading mortality cause in patients with cirrhosis. It is estimated that the main causes of liver disease leading to cirrhosis and/or HCC in Europe include hepatitis C (HCV) infection in 60% of patients, hepatitis B (HBV) infection in 15% of patients, and alcohol abuse in 10% of patients. The rest are attributed to metabolic liver diseases: nonalcoholic hepatic steatosis, alpha-1AT deficiency, hemochromatosis, and congenital tyrosinemia; carcinogens: aflatoxin B1, thorotrast, and dimethylaminoazobenzene; and some medications: anabolic hormones, estrogens, methyldopa, and methotrexate. The risk of HCC development increases with risk factor and cofactor accumulation; for example, cirrhosis concomitant with HBV infection increases this risk 1000-fold. The HBV/HCV, HBV/HIV, or HCV/HIV coinfection (or coinfection with all three types of a virus), alcohol abuse, long-term tobacco use, and other factors promote HCC development [2].

## 2. Primary Hepatocellular Carcinoma: Diagnostic Recommendations

The diagnostic management algorithms concerning cases of suspected cancerous lesions within the liver are (or at least should be) commonly known. They were developed and recently also updated by the European Association for the Study of the Liver (EASL) and the AASLD [3]. These are based (regardless of uncharacteristic clinical symptoms or their absence) on the findings of diagnostic imaging and histopathological assessment and in some cases on the serum alpha-fetoprotein (AFP) or des-gamma carboxyprothrombin (DCP) levels. The basis for an early diagnosis of HCC is regular screening of selected high-risk groups of patients. Patients with liver cirrhosis of variable etiology and Child-Pugh scores A and B as well as individuals with HBV infection and a family history of HCC need rigorous monitoring every 6 months. Liver ultrasound evaluation is recommended as a part of this monitoring. If the suspected focal lesion is 1-2 cm in diameter, 2 separate contrast-enhanced diagnostic imaging procedures must be performed (e.g., contrast-enhanced ultrasound, done relatively infrequently these days, contrast-enhanced computed tomography (CT) or triphasic, and contrast-enhanced magnetic resonance imaging (MRI) scans). If the findings are still inconclusive, the diagnosis should be confirmed with a cytological or histopathological evaluation. According to the international diagnostic guidelines, if the lesion is larger than 2 cm, HCC can be diagnosed based on contrast-enhanced imaging, confirming hypervascularization during the arterial phase and quick washout during the venous phase. If the histopathological evaluation fails to confirm cancerous lesion, diagnostic imaging should be performed repeatedly every 3–6 months.

The diagnosis of HCC can be based on AFP level, once the concentration exceeds 350 mg/dL. This parameter, however, was not included in the current screening programmes due to its low sensitivity in patients with smaller-size lesions. However, the consecutive increase of AFP level in a patient with liver cirrhosis should always raise the suspicion of hepatocellular carcinoma. The algorithms for HCC diagnosis are virtually unambiguous. Even though, due to the challenges faced in clinical practice, the diagnostic process is often delayed, which precludes early treatment and is reflected in the decreased survival.

## 3. Treatment Options

Until recently, there were no effective treatment methods available for HCC patients. However, a significant progress has been made in this respect in recent years. Complex management of HCC includes radical (surgery), conservative (medical), palliative, causal, and supplementary treatment. Psychological counselling, dietary interventions, and medical nutrition therapy belong to the supplementary treatment category. HCC can be treated successfully only if diagnosed early enough.

### 3.1. Radical Treatment

The only radical treatment offering some curative potential is complete surgical removal of cancerous lesion, that is, tumour excision with the adjacent liver tissue (partial hepatectomy and lobectomy—although these procedures do not address the underlying liver disease) or liver transplant surgery. Unfortunately, due to the advanced stages of HCC at the moment of diagnosis, only <20–30% of patients can benefit from surgical treatment. Surgery is possible in patients staged according to BCLC as very early (<2 cm) or early HCC, provided that the lesion is limited to 1 lobe only (i.e., liver function is normal and no signs of portal hypertension are observed). Small lesions are excised with 1 cm surgical margin. If the lesions are more extensive but no major vessel involvement is present, liver transplant surgery is indicated.

The alternatives to tumour resection are radiofrequency ablation (RFA), laser photoablation, microwave ablation (MWA), cryoablation, and percutaneous ethanol injection (PEI). Each procedure must be repeated several times, and treatment outcomes in small tumours (up to 2 cm) are comparable to those of surgical treatment.

### 3.2. Medical (Conservative)/Palliative Therapy

If a patient cannot be treated surgically, medical (conservative)/palliative therapy is used (stages A–C according to BCLC), which includes the following.

1. Transcatheter procedures are as follows: transarterial embolization (TAE), transarterial chemoembolization (TACE) (performed if resection cannot be performed or as a “bridging” procedure prior to liver transplant surgery), and radioembolization.

However, portal vein thrombosis or tumour infiltrating blood vessels preclude such interventions. The patient requires appropriate preparation and supplementary treatment during the perioperative period. These are high-risk procedures and 60–80% of patients develop some complications, which lead to death in 3% of cases. The estimated efficacy of such treatment ranges between 35 and 40%. However, a complete response can be achieved in less than 2% of cases [4].

(2) Intraoperative radiation therapy: brachytherapy and external beam radiation therapy (teletherapy): the available external beam techniques are intensity modulated radiation therapy (IMRT) and stereotactic radiation therapy; the efficacy is referred to as good local control and pain relief; treatment outcomes may be further improved by the simultaneous hepatic artery embolization [5].
(3) Neoadjuvant and adjuvant therapy: the beneficial effect of immune therapy or hormone replacement therapy on the efficacy of other HCC treatments has not been proved [6].
(4) Systemic treatment:
  - Chemotherapy:
    - Doxorubicin and cisplatin-based regimens, efficacy <10%.
    - Complex regimens: PIAF, XELOX, and GEMOX (gemcitabin plus oxiliplatin), efficacy <22%: chemotherapy of HCC is associated with high toxicity and numerous adverse effects, especially in patients with concomitant cirrhosis [7–10].
  - Angiogenesis inhibitors and cellular signalling pathway blockers: sorafenib is the only approved drug in this group; it acts on a cancer cell level and inhibits multiple kinases: tyrosine kinases (VEGFR2, PDGFR, c-KIT, and receptor) and serine-threonine kinases (b-raf and p-38); at the same time sorafenib blocks the RAF/MEK/ERK signalling pathways, inhibits tumour angiogenesis, and induces tumour cell apoptosis; the inclusion criteria for sorafenib treatment are A–C, PS 0–2, and Child-Pugh A-B scores; the total survival median increased by approximately 11 months (6–14 months) in 40% of treated patients; even superior outcomes were achieved in patients on combination therapy based on sorafenib + daunorubicin/ capecitabine /oxaliplatin; the survival median was increased by additional 8 months; when TACE is combined with sorafenib, the results are not unambiguous.
  - Hormone replacement therapy (androgenic and progesterone inhibitors): ineffective and not used.
  - Octreotide: its efficacy has not been proven yet.

According to current opinions, supported by the published evidence, antiviral treatment is a crucial element of complex HCC treatment (although antiviral medications do not exert any proven delay effect on tumour growth) and plays an important role in tumour spread and/or recurrence prevention in patients with HBV or HCV infections (as they constitute the most numerous group of HCC patients from the epidemiology perspective) [11–13].

HCC diagnosis can be particularly challenging in some cases and the obtained results seem ambiguous, which is illustrated by the three cases we present below.

## 4. Case Reports

### 4.1. Case Report 1

A 33-year-old male is diagnosed with HCV infection, genotype 3, in 1993. In 2000 histopathological evaluation of liver biopsy specimen showed severe inflammation (G3 level) and fibrosis (S3 level) as well as steatosis. The patient underwent recombined interferon and ribavirin treatment twice in 2001 and 2003. Both attempts to treat him failed, although his HCV genotype typically has a good prognosis for treatment response. The patient regularly attended follow-up visits. In 2010 a gradual increase of AFP level was observed: August 2010, 35.77 ng/mL, May 2011, 189.1 ng/mL, and September 2011, 4062.36 ng/mL, whereas the general health status of the patient was good and he did not display any clinical symptoms of complete cirrhosis. The ultrasound scans performed regularly at 6-month intervals and the contrast-enhanced abdominal CT scan performed in May 2011 did not show any cancerous lesions within the cirrhotic liver. Due to very high AFP levels and negative results of already performed diagnostic imaging procedures, the patient was admitted to our department in September 2011. The contrast-enhanced MRI was performed, which showed a single focal lesion sized 80 × 50 × 80 mm located within the 6th and 7th segment and suggestive of HCC. A core needle biopsy confirmed the diagnosis of HCC. Due to lesion size and patient's health which deteriorated rapidly, no conservative or antiviral treatment was commenced and the patient died two months later.

### 4.2. Case Report 2

**Figure 1:**
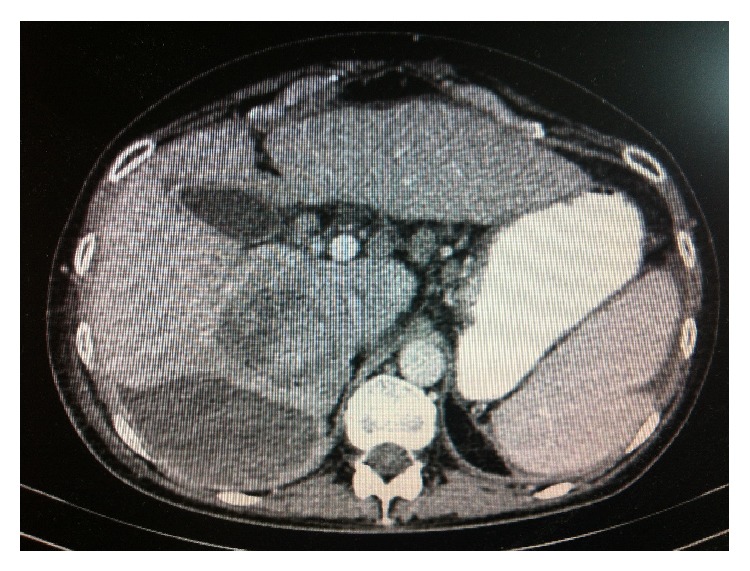
A three-phase CT of the abdomen. In a 4-segment focal lesion with typical enhancement in contrast phase and washout effect in a venous phase.

The histopathological evaluation of liver biopsy specimen of a 50-year-old female diagnosed in 2001 with HCV infection (G1b genotype) and with the history of HBV infection (particularly unfavourable situation from the perspective of HCC pathogenesis) showed moderate inflammation and fibrosis (G2, S2). The patient underwent a 48-week therapy with peginterferon-alfa combined with ribavirin in 2004. However, the treatment did not lead to the sustained virologic response (SVR). The follow-up biopsy performed in 2006 showed slight disease progression (G2-3, S2-3). Due to the concomitant thrombocytopenia, the patient was subsequently treated with natural interferon and ribavirin. The therapy, however, was discontinued after 12 weeks because of lack of early virologic response. The patient remained under the care of Infectious Disease Outpatient Clinic and the follow-up ultrasound scans were performed on a regular basis. In May 2010 the patient was admitted to our department due to the deterioration of her general health status, including significant weight loss and clinical symptoms of complete, compensated, active cirrhosis. The triphasic CT scan showed a lesion suggestive of HCC located within the 4th segment; however, due to the size of the lesion the patient was not qualified for surgery (Figure 1). The targeted fine needle biopsy did not confirm the malignancy. The follow-up ultrasound scans did not show the progression of the described lesion; the AFP level did not increase, either, and remained within the range of 36.89 ng/mL to 44.6 ng/mL. Due to significant diagnostic uncertainties, another (core needle) biopsy of the tumour was performed in September 2011, which confirmed the presence of cirrhosis. The patient was readmitted to our department 3 months later due to liver decompensation and she died in a month. The final diagnosis of HCC was made during an autopsy.

### 4.3. Case Report 3

A 65-year-old female with liver cirrhosis of mixed-aetiology (alcohol abuse + HCV G1b infection), confirmed in 1993 with histopathological findings in the biopsy specimen, was admitted to our department in June 2010 for the extensive diagnosis of a hepatic focal lesion revealed within the 6th segment in a CT performed in February 2008. For the undetermined reason the patient had not attended the recommended follow-up consults and imaging procedures for 2 years. At admission the Child-Pugh score was A5; the patient had concomitant COPD and was an active tobacco user. The ultrasound scan showed a normoechogenic focal lesion sized 21 × 12 mm, which had no distinct margins accompanied by the peripheral hypoechoic halo zone. The patient's serum AFP level was 10.96 ng/mL. For the abovementioned reasons, the patient was not immediately qualified for causal treatment of HCV infection (such patients are included in the antiviral treatment scheme at the moment). The contrast-enhanced abdominal CT scan performed 3 months later showed a heterogeneous abnormal focus sized 42 × 27 mm, localized within the 6th segment, which compressed the right portal vein branch. Two new lesions sized 8 mm each were additionally revealed within the 8th segment. Moreover, signs of portal hypertension were shown. As the findings were ambiguous, in order to distinguish cancerous lesion from the intrahepatic arterioportal venous malformation abdominal angio-CT scan was performed, as recommended by the radiologist. However, the findings were still inconclusive, so the diagnosis could not be made. At that time the patient did not give her consent to invasive diagnostic procedures. The patient was hospitalized for the second time in March 2011. This time her Child-Pugh score was 6 and the AFP level remained stable at 11.5 ng/mL. The contrast-enhanced MRI revealed a focal lesion of poorly defined polycystic margins within the 6th segment, sized 4.5 × 2.7 × 3.5 cm, and hepatic angioma and focal hepatic steatosis were excluded. The ultrasound-guided core needle biopsy did not confirm neoplastic malignant lesions or their precursors (dysplastic focal lesions). The image was typical of complete inflammatory cirrhosis, concomitant with alcoholic steatohepatitis and steatosis. The patient was readmitted to our department for follow-up imaging in October 2011. The AFP level decreased to 6.71 ng/mL. The ultrasound scan revealed a lesion sized 51 × 65 × 69 mm within the 6th segment. The lesion infiltrated the right hepatic vein, which was an explicit confirmation of its malignant nature. With clinical diagnosis of HCC the patient was referred to the Organ Transplant Surgery Outpatient Clinic. Unfortunately, the tumour had already spread to the abdominal blood vessels, so she could not be approved as a liver recipient. A subsequent CT-guided core needle biopsy and histopathological evaluation of the biopsy specimen were performed as a part of qualification for treatment with sorafenib. The evaluation did not show any areas of neoplasia or dysplasia. The patient died a few months later due to the generalized cancer.

## 5. Discussion

Early diagnosis of HCC is an obvious key to potential good treatment outcomes. But, unfortunately, 70%–80% of patients cannot benefit from radical treatment (i.e., liver resection and liver transplant) due to being diagnosed too late. The delayed diagnosis results in most cases from the lack of proper follow-up and regular diagnostic imaging. This can be jointly attributed to the low awareness of the disease among the GPs, high costs, and patients' neglect. Other reasons include old diagnostic equipment, inexperienced clinicians assessing the obtained scans, choice of improper imaging technique (e.g., plain CT/MRI), relying on normal AFP levels, and the lack of histopathological confirmation of the diagnosis. Unfortunately, mostly for technical reasons, histopathological diagnosis is not always possible. The abovementioned problems caused the delay or lack of in vivo diagnosis of HCC in the discussed cases precluding early therapeutic interventions. Diagnostic imaging constitutes the main category of diagnostic tools used in HCC. Ultrasound evaluation used to be and still remains the standard screening technique in primary hepatocellular carcinoma. If performed every 6 months by an experienced radiologist/clinician, the ultrasound scan enables detection of smaller-size lesions and, in turn, faster diagnosis and effective treatment [14]. Computed tomography is a very good diagnostic tool for HCC patients. However, in order to be useful it must be contrast-enhanced, four-phase scan (precontrast phase, arterial phase, portal venous phase, and delayed phase), which requires well-trained, experienced healthcare personnel. The abdominal, contrast-enhanced MRI offers superior sensitivity, if performed and interpreted by an experienced radiologist. Two types of contrast media are used for MRI: manganese- or gadolinium-based agents characterised by hepatocyte affinity or agents captured by the mononuclear phagocyte system. The main advantage of the discussed diagnostic methods includes the possibility to differentiate between hepatocellular and nonhepatocellular malignancies [15].

Liver biopsy with the subsequent histopathological evaluation of specimen still remains the diagnostic standard for chronic hepatitis and HCC. However, the efficacy of different types of biopsy in HCC differs; the targeted fine needle biopsy is estimated to be effective in 10–30% of cases, whereas the estimated efficacy of core needle biopsy is approximately 50%. Such low efficacy is caused not only by the improper biopsy technique, but rather by the tumour itself, which can be highly differentiated and may even contain the intact hepatic parenchyma. In order to improve the efficacy of biopsy the use of newer systems (e.g., Tru-Cut biopsy) seems to be reasonable.

The AFP level measurement has been used as a diagnostic marker of primary hepatocellular carcinoma since 1970s. The elevated AFP levels correlate with large lesions, over 5 cm in diameter. However, the AFP concentration typically remains normal if the lesion is small (<2 cm). The primary hepatocellular carcinoma, not associated with AFP level elevation throughout its entire course, constitutes a separate diagnostic challenge. That is why the EASL no longer recommends the use of AFP in patient screening for HCC. This biomarker is actually useful in the assessment of tumour reoccurrence, which is a separate topic for discussion.

Liver elastography appears to be an interesting diagnostic tool, although its use is limited; the researchers from Japan and the United Kingdom showed a high risk of HCC in patients with liver stiffness over 20 kPa [16] and the risk of postoperative tumour reoccurrence in patients with liver stiffness over 13.4 kPa [17].

## 6. Summary

1. Low sensitivity of ultrasound imaging and poor quality CT preclude early diagnosis of HCC and proper interventions, especially in smaller-size tumours.
2. The technical difficulties in obtaining specimens for histopathological evaluation (e.g., too small specimens obtained during the targeted fine needle biopsy, subdiaphragmatic or periportal lesions, significant comorbidities, etc.) preclude early histopathological diagnosis in many cases, thus delaying or precluding treatment.
3. The ambiguity of diagnostic imaging findings as well as long waiting time for hospital admission end imaging reports deprives many patients of their chance for an early, effective treatment.
